# Progesterone receptor blockade in human breast cancer cells decreases cell cycle progression through G2/M by repressing G2/M genes

**DOI:** 10.1186/s12885-016-2355-5

**Published:** 2016-05-23

**Authors:** Susan E. Clare, Akash Gupta, MiRan Choi, Manish Ranjan, Oukseub Lee, Jun Wang, David Z. Ivancic, J. Julie Kim, Seema A. Khan

**Affiliations:** Department of Surgery, Feinberg School of Medicine, Northwestern University, 303 E. Superior Street, Lurie 4–111, Chicago, IL 60611 USA; Department of Obstetrics and Gynecology, Feinberg School of Medicine, Northwestern University, 303 E. Superior Street, Lurie 4–111, Chicago, IL 60611 USA

**Keywords:** Progesterone receptor, Telapristone acetate, Breast cancer, Cell cycle, G2/M, Luteal, Antiprogestin

## Abstract

**Background:**

The synthesis of specific, potent progesterone antagonists adds potential agents to the breast cancer prevention and treatment armamentarium. The identification of individuals who will benefit from these agents will be a critical factor for their clinical success.

**Methods:**

We utilized telapristone acetate (TPA; CDB-4124) to understand the effects of progesterone receptor (PR) blockade on proliferation, apoptosis, promoter binding, cell cycle progression, and gene expression. We then identified a set of genes that overlap with human breast luteal-phase expressed genes and signify progesterone activity in both normal breast cells and breast cancer cell lines.

**Results:**

TPA administration to T47D cells results in a 30 % decrease in cell number at 24 h, which is maintained over 72 h only in the presence of estradiol. Blockade of progesterone signaling by TPA for 24 h results in fewer cells in G2/M, attributable to decreased expression of genes that facilitate the G2/M transition. Gene expression data suggest that TPA affects several mechanisms that progesterone utilizes to control gene expression, including specific post-translational modifications, and nucleosomal organization and higher order chromatin structure, which regulate access of PR to its DNA binding sites.

**Conclusions:**

By comparing genes induced by the progestin R5020 in T47D cells with those increased in the luteal-phase normal breast, we have identified a set of genes that predict functional progesterone signaling in tissue. These data will facilitate an understanding of the ways in which drugs such as TPA may be utilized for the prevention, and possibly the therapy, of human breast cancer.

**Electronic supplementary material:**

The online version of this article (doi:10.1186/s12885-016-2355-5) contains supplementary material, which is available to authorized users.

## Background

Endocrine agents are a mainstay of therapy for hormone receptor positive breast cancer. Pharmacologic antagonists targeting both estrogen and progesterone activity were developed in the 1960s [1]. In the ensuing half-century, selective estrogen receptor (ER) modulators (SERMs) and Aromatase Inhibitors (AIs) have had unequivocal success in the treatment and prevention of breast cancer [2–4]. The antiprogestin onapristone (ZK 98.299) showed preclinical and clinical efficacy but trial recruitment was halted secondary to significant liver toxicity largely attributable to binding to other nuclear receptors, most notably glucocorticoid receptor (GR) [5, 6]. Consequently, the strategy of blocking progesterone receptor (PR) activity to prevent and treat breast cancer was largely abandoned. However, there is compelling evidence to suggest that blocking PR signaling may have significant clinical utility. Data from the Women’s Health Initiative and the Million Woman Study clearly show that exposure to medroxyprogesterone acetate (MPA), a progestin, is a risk factor for the development of breast cancer [7, 8]. Progesterone may promote oncogenic progression by stimulating the proliferation that occurs during the menstrual cycle [9], by reanimating stem cells [10], or by driving the proliferation of early, i.e. occult, lesions [5].

The recent availability of relatively potent progesterone antagonists with little to no antiglucocorticoid activity, such as telapristone acetate (TPA; CDB-4124) [11, 12] prompts renewed interest in the anti-cancer effects of these agents. Competitive binding assays show that while TPA retains much of the antiprogesterone activity of mifepristone (RU-486), the antiglucocorticoid potency of TPA and its metabolites is less than 4 % that of mifepristone [11]. In an ongoing Phase II pre-surgical window trial, we are testing the anti-proliferative efficacy of TPA in early stage breast cancer (clinicaltrials.gov NCT01800422). In the present report, we have employed TPA as a tool to probe the actions of a variety of progestogens (progesterone, MPA, and R5020) in breast cancer cell lines. R5020 (promegestone) is a 19-norprogesterone derivative with a higher binding affinity for PR and a slower dissociation rate from the receptor-ligand complex when compared to progesterone [13, 14]. Additionally, we sought to identify a set of genes that signify progesterone activity or blockade. Our goal is to use these genes or combinations as biomarkers indicating successful abrogation of progesterone signaling in early phase trials that will test the utility of antiprogesterone therapy.

## Methods

### Cell culture and chemicals

T47D, BT474 and MCF-7 breast cancer cell lines were obtained from Dr. Charles V. Clevenger (Department of Pathology, Virginia Commonwealth University, Richmond, VA, USA) and MCF10A immortalized normal mammary epithelial cells were purchased from The American Type Culture Collection (ATCC, Manassas, VA, USA). T47D, BT474 and MCF-7 are ER+/PR+ cell lines; T47D has the highest PR expression of the three cell lines [15]. T47D, BT474 and MCF-7 cells were maintained in phenol free MEM supplemented with 10 % FBS (Atlanta Biologicals, Norcross, GA, USA), 2 mM L-glutamine, 1 % MEM-NEAA, 0.075 % Sodium bicarbonate and 100 units/mL of penicillin, 100 μg/mL of streptomycin and 25 μg/mL of Fungizone® in a humidified incubator at 37 °C and 5 % CO_2_. MCF10A cells were grown in DMEM/F12 containing 5 % horse serum, 20 ng/mL EGF, 0.5 mg/mL hydrocortisone (Sigma-Aldrich, St. Louis, MO, USA), 100 ng/mL cholera toxin (Sigma-Aldrich, St. Louis, MO, USA), 10 μg/mL insulin, and 100 units/mL of penicillin, 100 μg/mL of streptomycin, and 25 μg/mL of Fungizone®. Cell growth media and all of the cell culture supplements were purchased from Gibco® (Carlsbad, CA, USA) unless indicated. Estradiol (E2), progesterone (P4), 17α-hydroxy-6α-methylprogesterone acetate (MPA) and Mifepristone (RU486) were purchased from Sigma-Aldrich (St. Louis, MO, USA). Promegestone (R5020) was obtained from PerkinElmer (Santa Clara, CA, USA). 17α-acetoxy-21 methoxy-11β[4-N,N-dimethylaminophenyl]-19-norpregna-4,9-diene-3,20-dione (telapristone acetate, TPA; CDB4124) was provided by Repros Therapeutics (The Woodlands, TX, USA). E2, and progestogens (P4, MPA and R5020) were reconstituted in ethanol and TPA in DMSO. All solvents were cell culture grade and the working solutions were stored at −20 °C.

### Cell viability assay

The viability of T47D cells was evaluated by MTT assay according to the manufacturer’s instructions (Roche Life Science, Indianapolis, IN, USA). 5,000–10,000 cells were plated per well of a 96-well plate in 200 μL of growth media supplemented with 5 % charcoal-stripped FBS (CHS/FBS, Atlanta Biologicals, Norcross, GA, USA) and incubated for 24 h. These hormone-starved cells were then treated with 10 nM P4, 10 nM MPA, 10 nM R5020 ± TPA (0.1 μM, 1 μM) alone or in combination with 1 nM E2. Control cells received ethanol. Cell viability at 24, 48 and 72 h was determined by measuring metabolic activity of living cells as relative colorimetric changes. All experiments were repeated at least three times. Two-way analysis of variance (ANOVA) was used to determine the significant differences between treatments. The Bonferroni test was used to analyze multiple comparisons. All statistical tests were performed using GraphPad Prism (GraphPad Software, La Jolla, CA, USA).

### Proliferation and apoptosis

Apoptosis and Cell proliferation were examined using Annexin V (Molecular Probes, Thermo Fisher Scientific, Waltham, MA, USA, Cat# A23204) and Ki-67 (BD Biosciences, San Jose, CA, USA, cat# 561126) labeling respectively. T-47D cells were cultured in regular media as described above. At 80–85 % cell confluence, the cell cycle was synchronized by serum starvation. Following that, treatment with vehicle, R5020 (10nM), and R5020 with TPA (1 μM) for 0 h, 24 h, 48 h, and 72 h in 5 % charcoal stripped FBS, phenol red free MEM (Atlanta Biologicals, Norcross, GA, USA) was performed. The treated cells were then disassociated, counted, aliquoted in two sets and incubated with Annexin V or Ki-67 as per manufacturer’s recommendations. Cell cycle was analyzed using BD LSRFortessa flow cytometer (BD Biosciences, San Jose, CA, USA) and data analysis was performed using Graphpad Prism Ver 6.0 (San Diego, CA, USA). Two-way ANOVA was utilized to determine the significance of the differences over the time course of the experiments and Tukey’s test to determine significance between treatments at individual time points.

### Immunoblotting

3 × 10^5^ cells of T47D and BT474 were hormone-starved for 24 h. T47D cells were then treated with 10 nM R5020 for 24 h. BT474 cells were incubated with 1 nM E2 for 72 h, washed twice with growth media, and treated with 10 nM R5020 for 24 h. Cells were harvested and whole proteins extracted in RIPA buffer (Pierce, Rockford, IL, USA) including protease inhibitor cocktail and EDTA. Protein concentration was determined using the BCA Protein Assay Kit (Pierce, Rockford, IL, USA) and identical amounts of protein were separated in 10 % NuPAGE Bis-Tris SDS/PAGE Protein Gels (Invitrogen, Carlsbad, CA, USA) followed by transfer onto a polyvinylidene difluoride membrane (Invitrogen, Carlsbad, CA, USA). The membrane was probed with anti-PR antibodies (Santa Cruz Biotechnology, Paso Robles, CA, USA) followed by incubation with a secondary goat anti-mouse antibody (Pierce, Rockford, IL, USA). The blots were developed using the ECL Prime Western Blotting Detection Reagent (Amersham, Piscataway, NJ, USA). Anti-GAPDH antibodies (Santa Cruz Biotechnology, Paso Robles, CA, USA) were used for loading controls of proteins.

### Cell cycle analysis

Cell cycle distribution was examined by measuring the cellular DNA content using propidium iodide (PI) and flow cytometry. T47D cells, growing in the exponential phase were hormone-starved for 24 h in growth media containing 5 % CHS/FBS; and BT474 cells, after 72 h exposure to E2, were treated with 10 nM P4, 10 nM MPA, 10 nM R5020 ± TPA (0.1 μM, 1 μM) alone or in combination with 1 nM E2 for 24 h. After incubation, cell pellets were collected by centrifugation, washed twice with PBS, fixed in 70 % (v/v) ice-cold ethanol for 24 h at −20 °C and then stained with PI (50 μg/mL) containing RNase (100 μg/mL) and 0.1 % Triton X-100 for 30 min in the dark at 37 °C. Cell cycle was analyzed using BD LSRFortessa flow cytometer (BD Biosciences, San Jose, CA, USA) and FlowJo vX (FlowJo, LLC, Ashland, OR, USA).

### Measurement of PRE promoter activity

The PRE-luciferase reporter plasmid was a generous gift from Dr. Dean P. Edwards (Baylor College of Medicine, TX). T47D, BT474 and MCF-7 cells (1.2 × 10^5^ cells) were plated in a 24-well plate and hormone-starved for 24 h. Cells were then transfected with 0.8 μg of PRE-luc reporter plasmid along with phRl-TK (0.01 μg) Renilla control plasmid using Lipofectamine 2000 (Life technologies, Carlsbad, CA, USA) according to the manufacturer’s instructions. The transfected T47D cells were treated with 10 nM P4, 10 nM MPA, 10 nM R5020 ± TPA (10 nM, 100 nM, 1 μM) alone or in combination with 1nM E2. Control cells received ethanol and DMSO as vehicle. Cells were processed and the luminescence from firefly and Renilla luciferase was measured using the Dual-Luciferase® Reporter Assay System (Promega, Madison, WI, USA) and the Synergy HT microplate reader (BioTek, Winooski, VT, USA). The relative PRE- luciferase activity was expressed as the ratio of the firefly luciferase/Renilla luciferase unit (RLU).

### Microarray analysis and statistical analysis

Three separate T47D cell cultures were used for microarray analysis. The experimental treatments were vehicle, 10 nM R5020, 1 μM TPA, and 10 nM R5020 with 1 μM TPA. All RNA samples were processed at the Genomics Core Facility in the Center for Genetic Medicine at Northwestern University (Chicago, IL). The quality of total RNA was evaluated using the Bioanalyzer 2100 (Agilent Technologies, Inc., Santa Clara, CA, USA). 150 ng of each RNA sample, with 260/280 and 28S/18S ratio of greater than 1.8, was used to make double-stranded cDNA. Gene expression analysis was performed using the Illumina Human HT-12v4 Expression BeadChip. Quality checks and probe level processing of the Illumina microarray data were further made with the R Bioconductor package lumi (http://www.bioconductor.org/packages/release/bioc/html/lumi.html). Data was quantile normalized, and hierarchical clustering and Principal Component Analysis were performed on the normalized signal data to assess the sample relationship and variability. Probes absent in all samples were filtered out according to Illumina’s detection p-values in the downstream analysis. Differential gene expression between the different conditions was assessed by a statistical linear model analysis using the bioconductor package limma (http://www.bioconductor.org/packages/release/bioc/html/limma.html). The moderated t-statistic p-values derived from the limma analysis above were further adjusted for multiple testing by Benjamini and Hochberg’s method to control false discovery rate (FDR) [16]. The lists of differentially expressed genes were obtained by the FDR criteria of <5 % and fold change cutoff of > ± 1.5. Data obtained from the microarray was further analyzed by MetaCore (Thompson Reuters; https://portal.genego.com) and Ingenuity Pathway Analysis (IPA; Qiagen, http://www.ingenuity.com).

### Validation of gene expression for selected 16 genes

Cell cycle regulating genes responding to both R5020 and TPA (microarray data) were compared with cell cycle genes upregulated by progesterone in luteal phase of normal breast tissue (RNA-Seq data) [17] and 16 genes that were significantly differentially expressed were identified. The expression of these 16 genes was validated with reverse transcription-quantitative polymerase chain reaction (RT-qPCR). Briefly, RNA from the gene arrays was reverse transcribed into cDNA using the SuperScript VILO cDNA Synthesis Kit (Life technologies, Carlsbad, CA, USA). Real-time qPCR was performed using an ABI PRISM 7900 Sequence Detection System (Applied Biosystems, Life technologies, Carlsbad, CA, USA). The geometric mean of housekeeping gene (GAPDH and β-Actin) was used as an internal control to normalize the variability in expression levels. PCR primers used for real-time PCR were purchased from integrated DNA technologies (Coralville, IA, USA) and the list of the primers is provided in Additional file 1: Table S4. Expression data of the 16 genes was normalized to housekeeping genes GAPDH and β-Actin to control the variability in expression levels and were analyzed using the 2^-ΔΔCT^ method described by Livak and Schmittgen [18]. The expression of the 16 genes was validated by real-time PCR using T47D and MCF10A cells. 6.0 × 10^5^ cells of T47D and MCF10A were hormone-starved for 24 h. Cells were then treated with 10 nM P4, 10 nM MPA, 10 nM R5020 ± TPA for 24 h. Vehicle treated cells were used as a control. Total RNA from samples was extracted using Trizol reagent (Life technologies, Carlsbad, CA, USA). 2 μg of total RNA was converted to cDNA using SuperScriptVILO master mix (Life technologies, Carlsbad, CA, USA) according to the manufacturer’s instruction. Real-time PCR and data analysis were as above. Two-way analysis of variance (ANOVA) was used to determine the significant differences between treatments. The Sidak correction was applied to analyze multiple comparisons. All statistical tests were performed using GraphPad Prism (GraphPad Software, La Jolla, CA, USA).

### Regulation of expression of the selected 16 genes

Motif analysis was performed using HOMER (v4.8) to identify common sequences in the promoters among the 16 genes of interest (Salk Institute, La Jolla, CA, USA; http://homer.salk.edu/homer/). The ENCODE transcription factor (TF) binding site tracks were enabled for the MCF-7 cell line to determine if promoters of the selected 16 genes are bound by the same TFs (https://www.genome.ucsc.edu/ENCODE/).

## Results

### Effect of progestogens and TPA on cell number

**Fig. 1:**
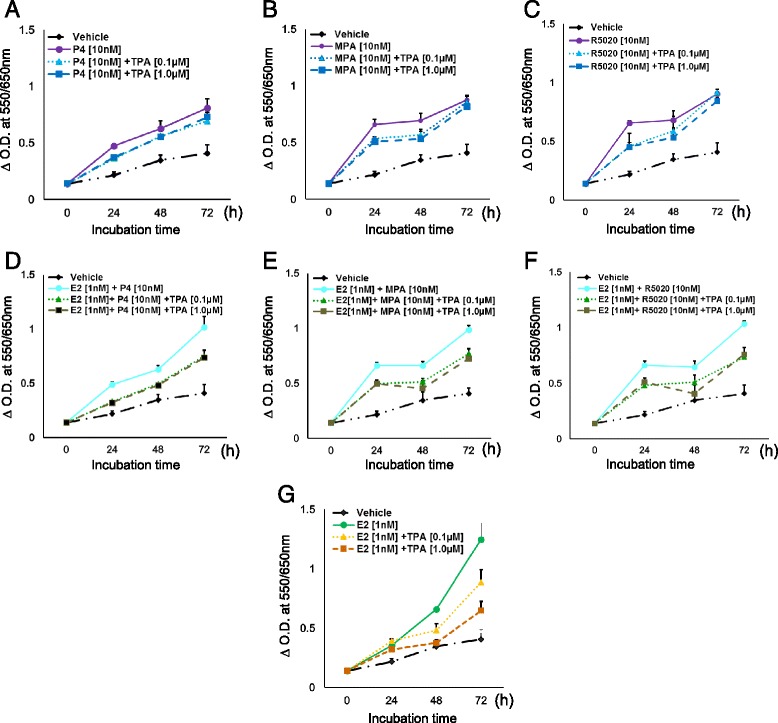
Determination of cell viability by MTT assay. T47D cells were hormone-starved for 24 h and treated for 24, 48, and 72 h with (**a**) P4 ± TPA, (**b**) MPA ± TPA, (**c**) R5020 ± TPA alone, or in combination with E2 (**d, e**, and **f**). Cells were also treated with E2 ± TPA (**g**). Vehicle treated cells were used as a control. X-axis: 24, 48, and 72 h time points. *p*-values for the various comparisons are provided in Additional file 2: Table S1

The proliferation of T47D cells was assayed in the presence of progestogens alone (P4, MPA and R5020) at 24, 48 and 72 h. There was significant stimulation of proliferation by all progestogens at 24 h as shown in Fig. 1a-c (Additional file 2: Table S1). Proliferation at 24 h was 2.1-fold greater in the presence of P4, and 3-fold greater in the presence of MPA (Fig. 1b) and R5020 (Fig. 1c) than with vehicle treatment. The proliferation of the MPA and R5020 cultures plateaus between 24 and 48 h; proliferation resumes between 48 and 72 h (Fig. 1b-c). The plateau is well known phenomenon in the setting of continuous progestogens and is due to arrest in late G1 consequent to increased levels of p21 and p27^kip^, and decreased levels of Cyclins A, B and D [19]. The increased formazan observed at 24 h in the presence of progestogens was blocked by the addition of the anti-progestin TPA; up to 30 % inhibition was produced by both low (0.1 μM) and high (1.0 μM) concentrations of the inhibitor (*p* < 0.001).

At 24 h, proliferation stimulated by E2 alone was less when compared to P4 alone (Fig. 1g and a); the combination of E2 with the progestogens mimicked the proliferation curves of the progestogens alone and there did not appear to be an additive or synergistic effect. However, at 72 h, proliferation in the presence of E2 alone (Fig. 1g) was 28–35 % greater than that of E2 plus the progestogens (*p* < 0.0001; Fig. 1d-f). The addition of TPA to E2 plus progestogen cultures resulted in 22–37 % inhibition of formazan production in comparison to E2 plus progestogens (*p* < 0.0001; Fig. 1d-f). The incremental decrease in formazan at 72 h, E2 vs. E2 + R5020 vs. E2 + R5020 + TPA, is observed best in 1 F. As judged from Fig. 1a-f, it appears that the major effect of TPA occurs in the first 24 h; after this time point the slopes of the lines between 24–48 h and 48–72 h are quite similar when E2 is present (Additional file 1: Table S2); the lines converge at 72 h when E2 is not present. Thus the effect TPA in T47D cells is more persistent in the presence of E2 + progestogens, than with progestogens alone (Figs A-C compared to D-F). To complete the picture, formazan production was measured in the presence of E2 and TPA but without progestogens. As shown in Fig. 1g, a dose dependent decrease occurs at both 48 (0.1 μM: 27 %; 1 μM: 43 %) and 72 h (0.1 μM: 29 %; 1 μM: 48 %), *p* < 0.0001 [20, 21]. Overall, the proliferation of T47D cells is most significant within the first 24 h after exposure to PR ligands alone or in the presence of E2, which is diminished by the addition of TPA at both high and low dose.

### Effect of progestogens and TPA apoptosis and proliferation

**Fig. 2:**
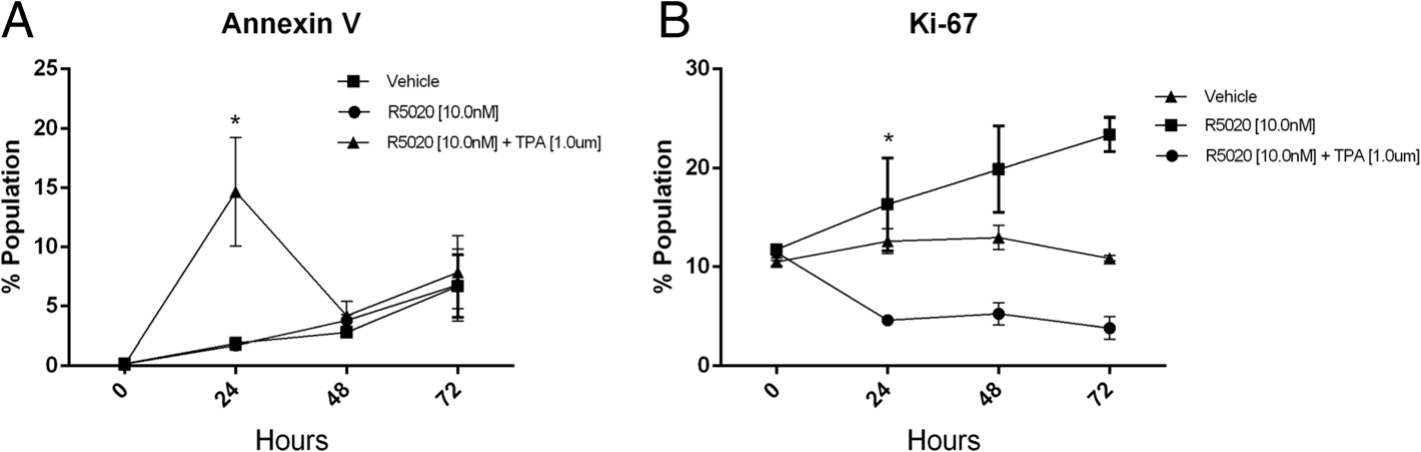
Annexin V and Ki67 expression analysis by flow cytometry. T47D cells were serum-starved for 24 h and treated with R5020 ± TPA for 24, 48 and 72 h. The percent of cells expressing each of the proteins was determined using flow cytometry. **a**. Annexin V. **b**. Ki67. Vehicle-treated cells were used as a control. * represents p value <0.05. h = hours

T47D cells cultured in the presence of R5020 [10nM] and TPA [1.0 μM] demonstrate a significant increase in apoptosis at 24 h (*p* < 0.05), which then decreases and is not different from to that of vehicle and R5020 at 48 and 72 h (Fig. 2a). Proliferation, as measured by Ki67, increased steadily and at a similar rate over the time course of the experiment in the presence of R5020 (Fig. 2b). The addition of TPA significantly decreased the percent of proliferating cells at 24 h (*p* < 0.05) and this percentage remained largely unchanged at the latter two time points.

### Effects of progestogens and TPA on the cell cycle

**Fig. 3:**
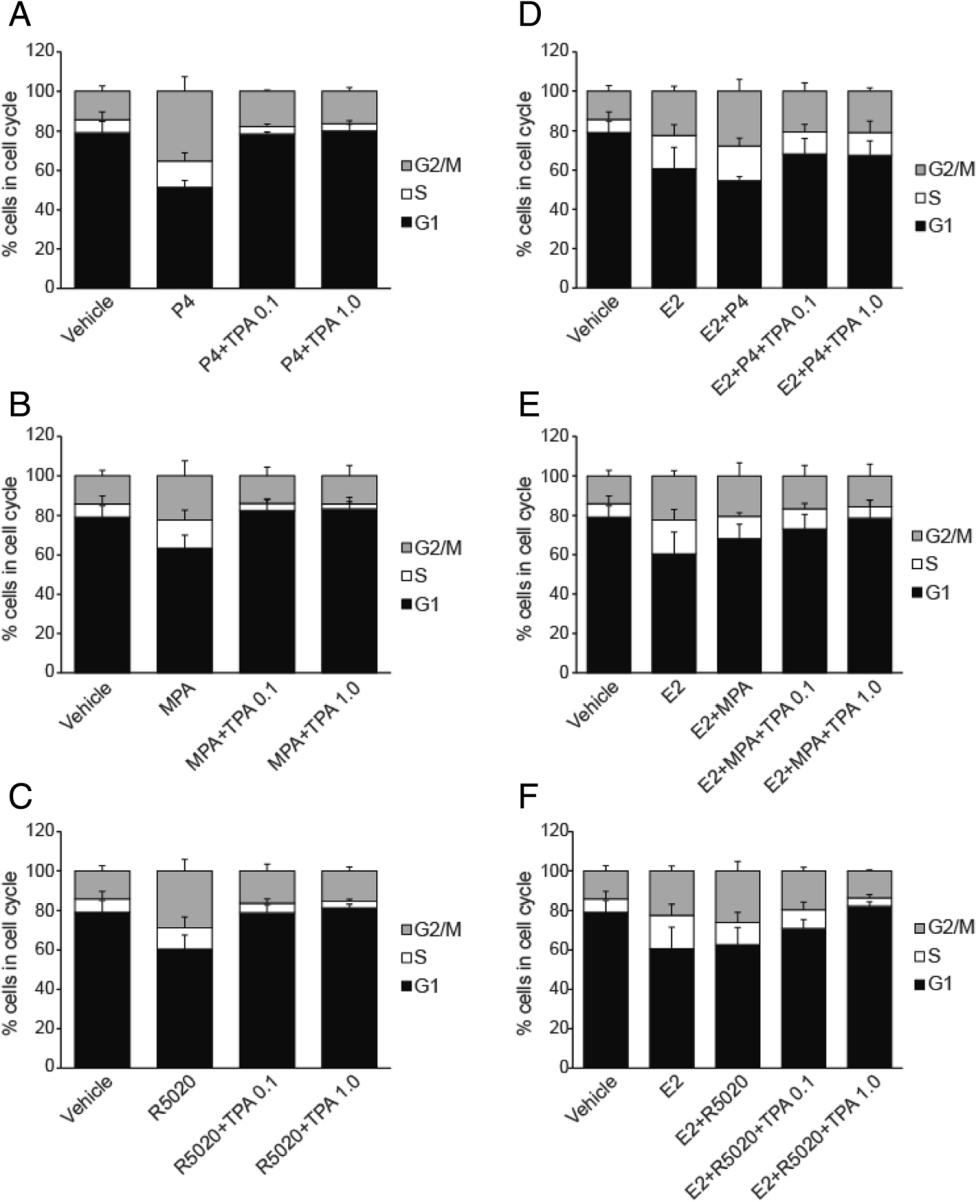
Cell cycle analysis by flow cytometry. T47D cells were hormone-starved for 24 h and treated with progestogens (P4, MPA, R5020) ± TPA (**a, b**, and **c**) and in combination with E2 (**d, e**, and **f**) for 24 h. The fraction of cells in G1, S and G2/M phase was determined by flow cytometry using Propidium iodide. Vehicle-treated cells were used as a control

Since majority of stimulation of proliferation of T47D occurs within the first 24 h after treatment with progestogens (P4, MPA and R5020) and this stimulation is blocked by TPA, the 24-h time point became the focus of further studies. Cell cycle analysis was performed after treatment of the cells with the progestogens ± TPA. As shown in Fig. 3a-c, P4, MPA and R5020 decreased the fraction of cells in G0/G1 and increased the fraction in G2/M and, to a lesser extent, S phase, when compared to vehicle at 24 h. The addition of TPA at both low and high doses (0.1 μM and 1 μM) resulted in increased numbers of cells in G0/G1 and decreased S and G2/M fractions (Fig. 3a-c). The addition of E2 alone resulted in fewer cells in G0/G1 and an increase in the fraction of cells in S and G2/M (Fig. 3d-f). Addition of TPA to E2 + P4 and E2 + R5020, at both low and high doses, produced an increase of cells in G0/G1 (Fig. 3d,f); however, low dose TPA did not affect cell cycle progression in E2 + MPA treated cells. Similarly, as shown in Fig. 3d-f, the percentages of cells in S and G2/M were decreased in the presence of both low and high dose TPA with E2 and P4 or R5020 but MPA showed no significant changes at the low dose of TPA.

**Fig. 4:**
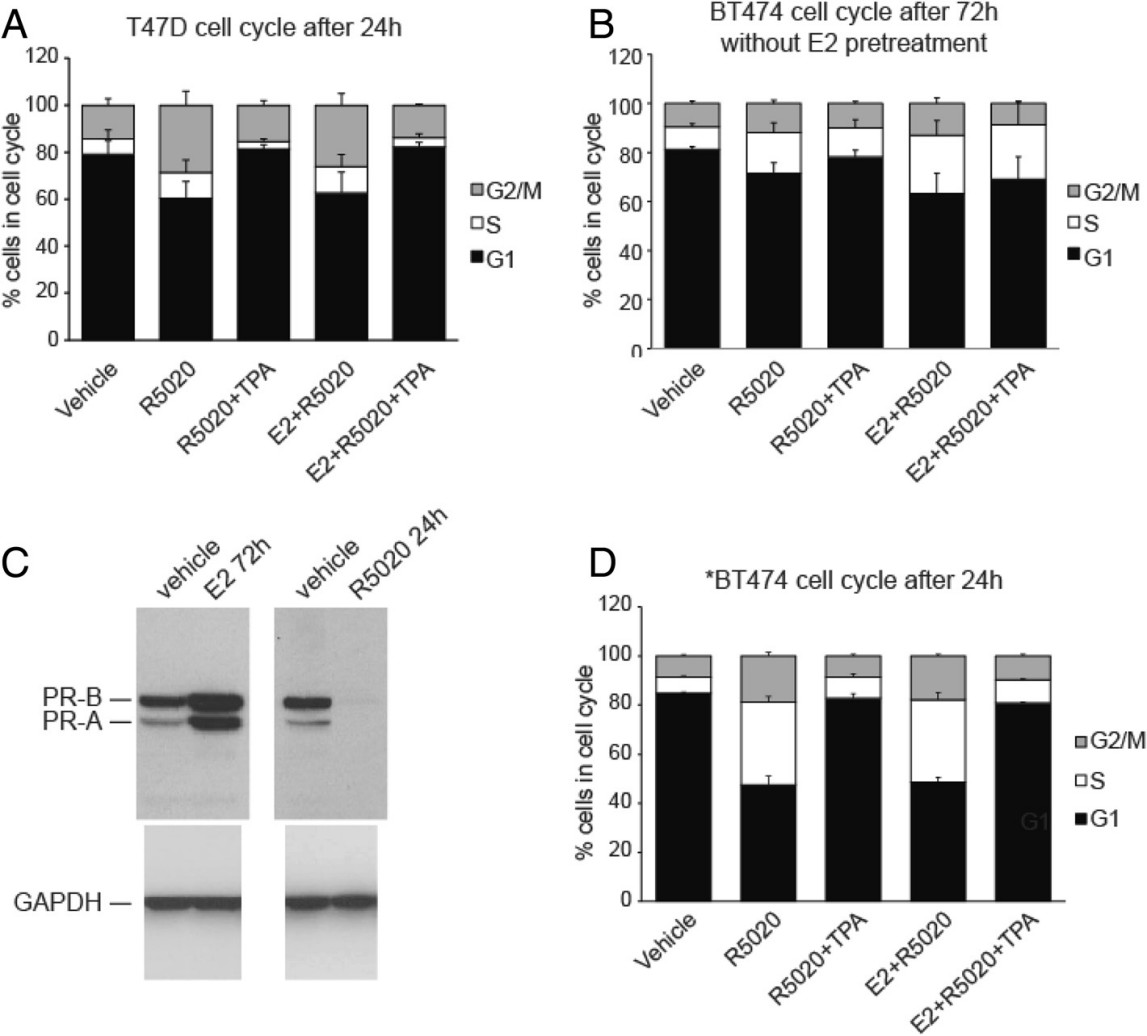
Cell cycle of T47D cells and BT474 cells after treatment with R5020 [10nM] or E2 [1nM] + R5020 [10nM] alone or in presence of TPA [1 μM]. **a**. T47D and **b**. BT474 cells were serum-starved for 24 h and subsequently treated with E2, R5020 and the antiprogestin TPA in various combination as indicated in figure for 24 h. Cell cycle analysis was performed in presence of Propidium Iodide to measure G1, S and G2/M fractions. **c**. Immunoblot of increased PR expression after 72 h of exposure of BT474 cells to E2 (left) and after 24 h of exposure to R5020 (right). E2 significantly increase both PR-A and B protein expression. The loss of PR expression with exposure to R5020 is indicative of high transcriptional activity and rapid protein turnover [44]. The blot has been cropped to remove the 48 h data. **d**. *BT474 cells were stimulated with E2 [1nM] for 72 h prior to treatment of R5020 and TPA to increase PR expression

The above experiment was repeated in a second cell line: BT474. In comparison to T47D, BT474 cells express less PR [15] and the response to R5020 was somewhat attenuated (Fig. 4a,b). Therefore, the BT474 cells were incubated with E2 for 72 h to increase PR expression (Fig. 4c) prior to treatment with progestogens and TPA. As shown in Fig. 4d, R5020 decreased the fraction of cells in G0/G1 and, in distinction to T47D (Fig. 4a), increased the fraction in S and, to a lesser extent, G2/M when compared to vehicle at 24 h. The addition of TPA to R5020 treatment resulted in increased G0/G1 and decreased S and G2/M fractions when compared to R5020 alone. In both T47D and BT474 cells, the addition of E2 to R5020 had no marked effect on the distribution of cells within the cell cycle compared to R5020 alone. Combining TPA with E2 and R5020 abrogated the effects on cell cycle progression in both cell lines.

### TPA blocks PRE reporter activity

**Fig. 5:**
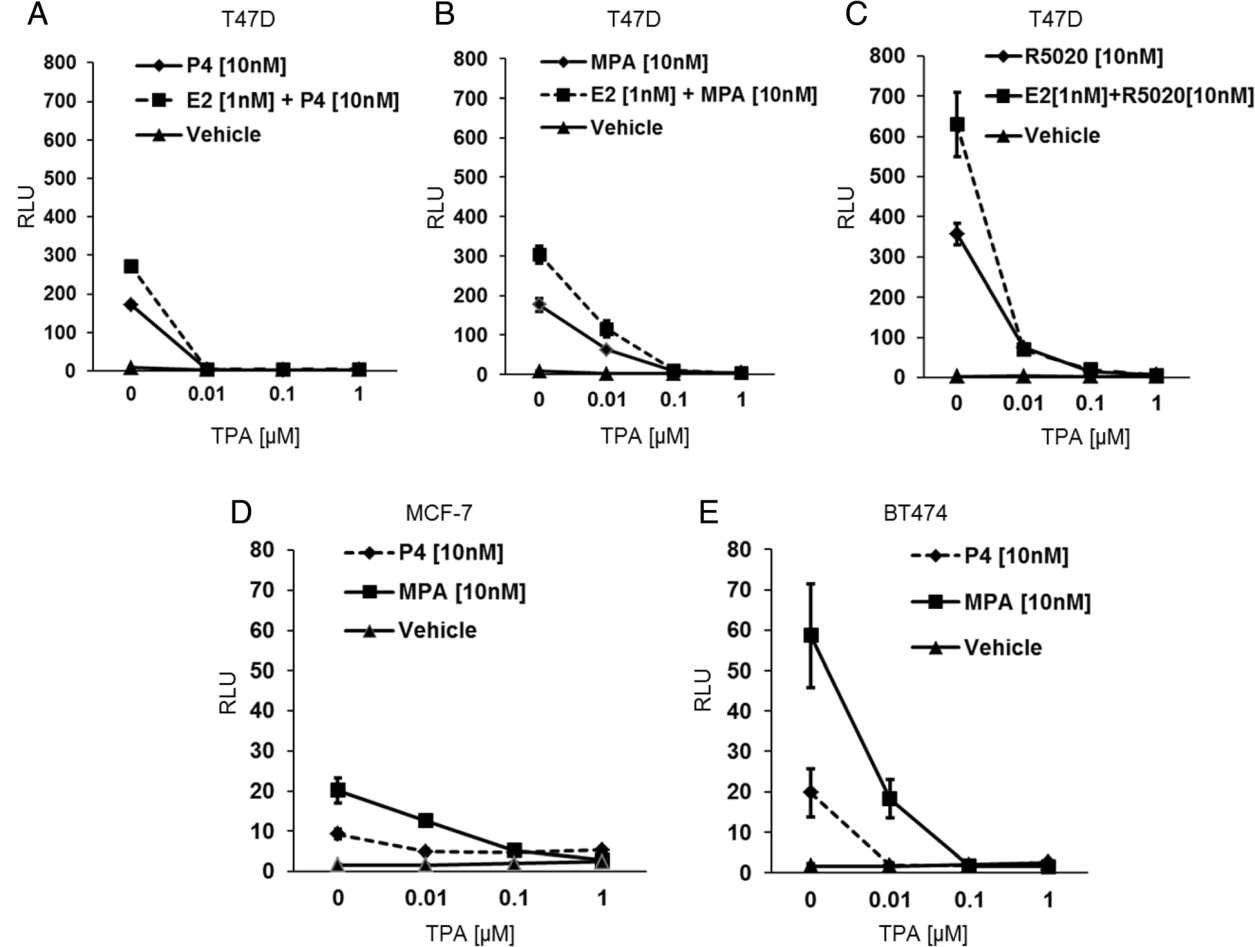
PRE promoter activity analysis by Dual luciferase assay. T47D, BT474, and MCF-7 cells were hormone-starved for 24 h and transfected with PRE-luc reporter plasmid along with phRl-TK Renilla control plasmid. The transfected T47D cells were treated with P4 (**a**), MPA (**b**), or R5020 (**c)** ± TPA (10nM, 100nM, 1 μM) alone or in combination with E2 (1nM). The transfected MCF-7 (**d**) and BT474 cells (**e**) received P4 or MPA ± TPA (10nM, 100nM, 1 μM). Luciferase activity was quantified using the Dual- Luciferase Reporter Assay Kit. The relative PRE- luciferase activity was expressed as the ratio of the firefly luciferase/Renilla luciferase unit (RLU)

Upon treatment with P4, MPA and R5020, PRE reporter activity increased significantly, which was further enhanced by the addition of E2 (Fig. 5a-c). T47D cells exhibited significantly higher induction of PRE, in comparison to MCF-7 (Fig. 5d) and BT474 (Fig. 5e). Increasing doses of TPA decreased the progestin-driven PRE reporter activity in a dose dependent manner. TPA effectively blocked P4-driven reporter activity at 10nM whereas R5020 and MPA driven reporter required 100nM for complete inhibition of activity. Similarly, TPA led to dose dependent inhibition of PRE induction in MCF7 or BT474 as shown in Fig. 5d and e, respectively. In summary, these data suggest TPA disrupts the recruitment or binding of ligand-bound PR at the PRE within the promoter region of progesterone-regulated genes.

### Identification of progestin-driven genes inhibited by TPA

**Fig. 6:**
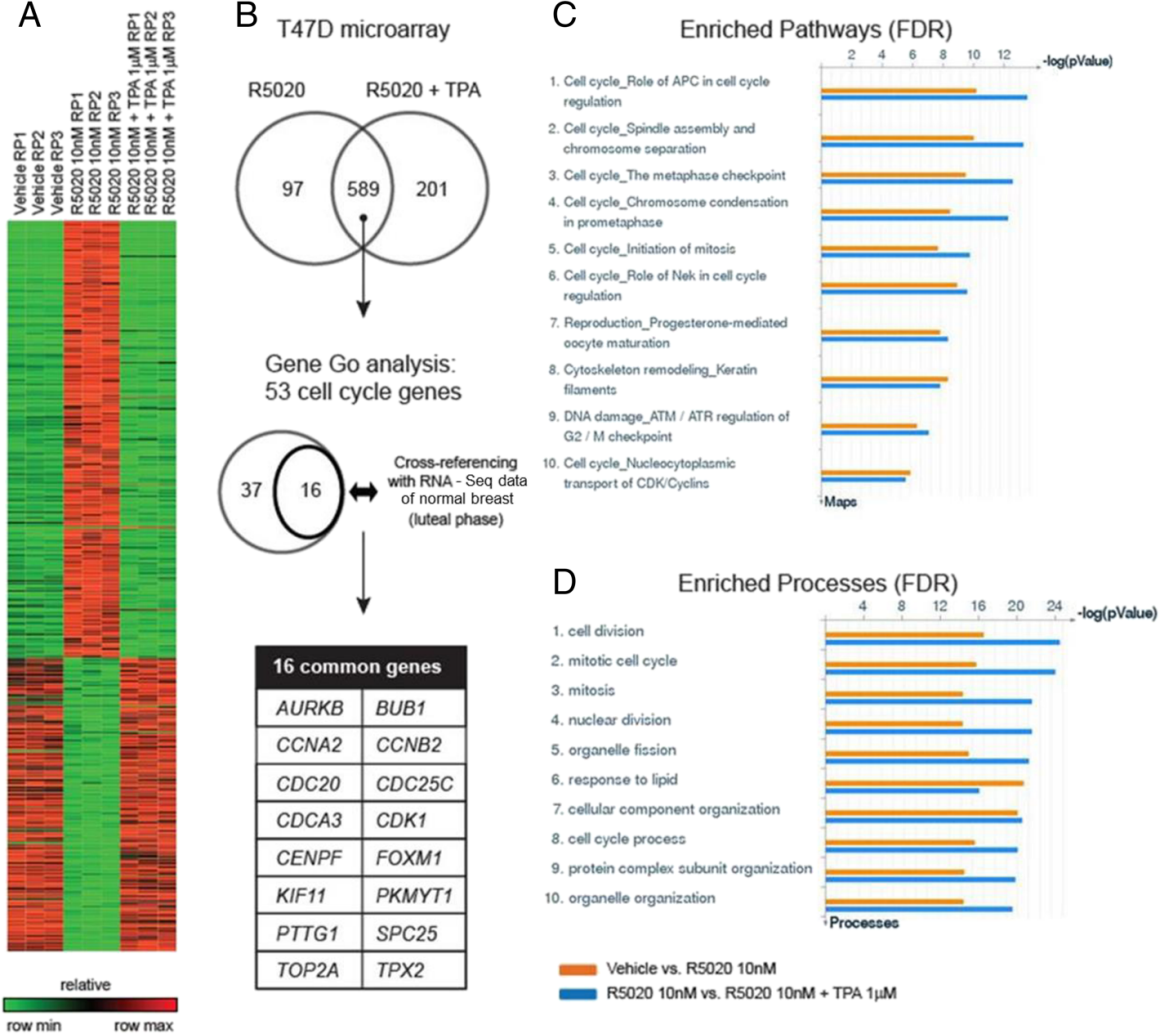
Analysis of gene expression microarray. T47D cells were treated with R5020 (10nM) ± TPA (1 μM) for 24 h. Vehicle treated cells were used as a control. Differential gene expression was assayed using the Illumina platform. (**a**) Heatmap of 589 genes commonly regulated by R5020 and TPA. (**b**) Identification of 16 cell cycle genes upregulated by progesterone both in normal and breast cancer cells. (**c**) Top ten enriched pathways for control vs. R5020 and R5020 vs. R5020 + TPA analyzed by GO. (**d**) Top ten enriched cell processes for control vs. R5020 and R5020 vs. R5020 + TPA

T47D cells were treated with 10nM R5020 for 24 h; vehicle-treated cells were used as control. A total of 686 genes were differentially expressed in presence of 10 nM R5020 (adjusted p value <0.001; Additional file 1: Table S2). Addition of TPA resulted in 790 genes that were differentially expressed compared to R5020 alone. Within these two gene sets there was an overlap of 589 genes, in that genes evincing increased expression with R5020 (≥1.5x) were decreased (≤1.5x) by the addition of TPA (Fig. 6b). The expression data was analyzed using MetaCore Gene Go (Thompson Reuters). Pathway enrichment analysis revealed that the pathways upregulated by the progestin R5020 are the same pathways downregulated by the addition of the antiprogestin TPA (Fig. 6c). These pathways are involved in the regulation of functions that occur during the cell cycle. The most significantly enriched cell processes are shown in Fig. 6d. In concert with the pathway data, the biologic process data revealed enrichment for mitosis, cytokinesis processes, organelle duplication and the cell cycle.

There were only six genes differentially expressed in the comparison of T47D cells treated with TPA alone versus control (data not shown).

### Progesterone receptor signaling and the G2/M phase of cell cycle

**Fig. 7:**
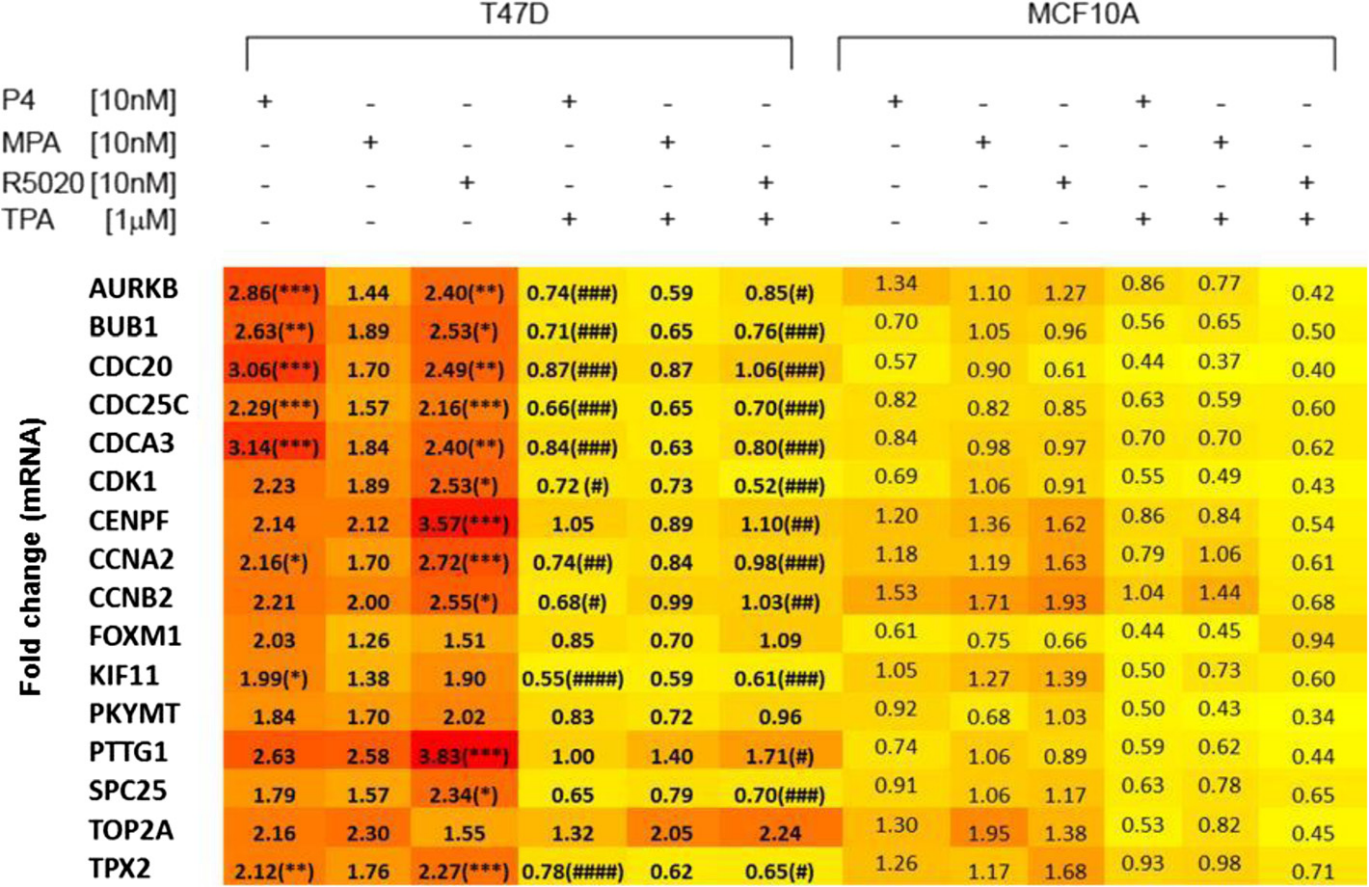
RT-qPCR validation of array data. RT- qPCR data for the sixteen genes show is displayed as a heat map (low to high: yellow to red) with fold-change in mRNA expression within the boxes. Hormone-starved T47D and MCF10A cells were treated for 24 h with Progesterone (P4), Medroxyprogesterone acetate (MPA), or Promegestol (R5020) alone or in combination with telapristone actetate (TPA) as indicated above the map. There were six independent repeats of the experiment. */**/*** represent p-values of < 0.5/<0.01/<0.001, respectively, for R5020 vs. vehicle; and #/##/### represent p-values of <0.5/<0.01/<0.001 for R5020 vs. R5020 + TPA

In order to cull the hundreds of differentially expressed genes for the purpose of identifying a set of genes that predicts functional progesterone signaling in human breast tissue, and to increase relevance to the prevention arena, genes regulated by R5020, as determined by the microarray (Additional file 1: Table S3), were compared with the genes which were significantly increased during the luteal (progesterone rich) phase in our RNA-Seq study [17]; 16 genes common to both gene sets were selected (Fig. 6b). Of note, the menstrual phase determinations in the RNA-Seq study were based on both menstrual dates and serum hormone concentrations. This strategy ensured that we were focusing on genes that are expressed in the normal breast consequent to progesterone stimulation. The majority of the 16 genes that emerged from this comparison are expressed during the G2/M phase of cell cycle. Additional analysis of the microarray data showed that the expression of the sixteen genes was significantly decreased, relative fold change <1.5 with adjusted p value <0.001 (Additional file 1: Table S3), by the addition of TPA to R5020. Technical validation (Additional file 3: Figure S1) was done by RT-qPCR using RNA from the microarray, which revealed significant upregulation of 13 genes by R5020 and an inhibition of this induction with TPA. Furthermore, this 16-gene panel was validated in an independent set of experiments (biologic validation) treating T47D or MCF10A cells with the three progestogens with or without TPA, using RT-qPCR (Fig 7). While all 16 genes evidenced increased expression in the presence of P4, R5020, and MPA, the levels of induction varied depending on the progestogens used. R5020 increased expression of the 16 genes, as did P4, however the induction was not as robust with MPA. TPA decreased expression of these genes regardless of the progestogens used. Topoisomerase 2A was an outlier in that its expression increased in the presence of R5020 and R5020 + TPA. MCF10A cells, which lack the expression of both ER and PR, demonstrated little to no response to the progestogens and TPA.

### Regulation of the expression of the 16 genes

Motif analysis 400 bp upstream of the transcription start site (TSS) and 100 downstream revealed the presence of the CHR motif for 11 of the 16 genes (Additional file 4: Tables S7 & S8). Likewise, the NFY motif was present in 14 of the 16 genes (Additional file 4: Tables S7 & S9). The MMB (Myb-MuvB) complex and FOXM1 have been demonstrated to bind to the conserved CHR element in 11 of the 16 genes (Additional file 4: Table S10) [22]. Ingenuity Pathway Analysis Upstream Analysis of the R5020 versus R5020 + TPA differentially expressed gene data displays inhibition of genes that are transcribed in response to the transcription factors PGR, FOXM1 and MYC (Additional file 4: Table S5). This analysis also predicted that NFYA and MYBL2 are inhibited in the presence of TPA although their differential expression did not meet our cut off of ± 1.5x. The SMARCE1 transcription factor was predicted to be activated. The TFs assayed as binding MCF-7 in the ENCODE data sets are relatively few. There was robust E2F1 binding of the majority of the 16 genes and MYC binding 11 of the 16 (Additional file 4: Table S6).

### Specific gene expression changes with mechanistic implications for TPA’s effects

*EGFR* and *p21* expression were downregulated by TPA, −1.40 and −2.61-fold respectively. A number of genes that encode proteins involved in chromatin remodeling have altered expression following the administration of TPA including *MSK1* (−1.67-fold), *SMARCE1* (1.63-fold), and *BAF57* (+1.63-fold).

## Discussion

We have described, for the first time, the molecular consequences of blocking progesterone signaling in PR positive breast cancer cells using a potent PR antagonist, TPA. Our major findings include the observation that blockade of progesterone signaling by TPA results in a decreased G2/M fraction, caused by decreased expression of genes that facilitate the G2/M transition. This effect is observed with P4 and R5020 and to a lesser extent with MPA. The addition of E2 to progestogens (P4, R5020, and MPA) results in somewhat greater increase in proliferation and more marked inhibition by TPA. In the absence of E2 (Fig. 1a-c) T47D proliferation at 72 h is unaffected by the presence of TPA. Progestin treatment of T47D cells leads to the rapid degradation of PR in the 26S proteasome [23], which suggests that the lack of drug effect in the absence of E2 may be due to the lack of a target. Pretreatment ER+/PR+ breast cells lines with estrogen for 72 h prior to the administration of a progestin had been shown to increase PR occupancy on DNA consequent to the increase in steady state levels of PR and the sites occupied are, to a great extent, the canonical PR binding sites [24]. The data from the E2 pretreated BT474 cells (Fig. 4d) contributes corroborating evidence that E2 driven expression of PR provides the target for the antiprogestin. The fact that the anti-proliferative efficacy of TPA requires the presence of E2 and P4 is highly relevant to the human condition, since humans are not exposed naturally to progestogens alone. TPA competes with progestogens for PR binding [11]. The PRE reporter experiments suggest that both MPA and R5020 have greater binding affinity for the receptor than P4 as it takes an order of magnitude greater concentration of TPA to have the same effect.

Groshong et al. studied the effect of R5020 ± mifepristone on T47D cells that are PR negative or contain one of the two PR isoforms [19]. With regard to cell cycle distribution, their data suggest that, for the most part, antiprogestins block the transient increase in mitogenic activity, i.e., the increase in S + G2/M, which peaks approximately 20–24 h after in the addition of the progestogen. For the PR-B isoform, there is no change in the G0/G1 fraction in the presence of mifepristone when compared to control arguing against an increase in quiescent, G0, cells; for the PR-A isoform there is an increase in this fraction. This is quite similar to what was observed for TPA: The majority of the effect on cell cycle distribution is due to the elimination of the increase in the S + G2/M fraction observed in the presence of progestogen alone but an increase in number of cells in G0 (Fig. 2) also probably contributes to a small increase in the G0/G1 fraction.

Our data add to the body of knowledge of progestogen signaling by providing a detailed view of the effects of longer, i.e., 24 h, exposure to R5020. Previously published data reflects exposure of T47D cells to R5020 for 6 h [18] and 12 h [25]. Many of the genes upregulated at 6 h are those associated with the rapid signaling via the cytoplasmic kinases, a process initiated by growth factor binding to its receptor with consequent regulation of the G1/S transition of the cell cycle (Additional file 5: Figure S2) [18]. Distinct from these data, our 24-h data reveal a preponderance of genes involved in mitosis and the G2/M transition (Additional file 5: Figure S2). Common to the 6 h and 24 h data sets, and data from normal breast during the luteal phase [17], is the upregulation of *c-MYC*, a progesterone target gene, which likely accounts for many of the effects on the cell cycle genes that were observed (Additional file 6: Figure S3). This is the subject of future experiments.

The transition into G2/M is governed by cyclin B/CDK1 [26]; expression of both of these genes is increased by R5020 binding to PR, and TPA significantly decreases their expression. Also decreased is the expression of the cell division cycle 25C (CDC25C) and CDC25B genes. The CDC25 proteins dephosphorylate CDK1, which promotes the G2/M transition [26]. The decrease in cyclin B1 and B2 RNA expression is likely secondary to TPA’s disruption of progestin/PR-induced classical transcriptional regulation of c-MYC expression [27]. The association of ERα-PR on the promoter of CCND1 and MYC drives the progestin (MPA)-induced expression of these genes and cell proliferation [28]. There is abundant evidence that c-Myc binds to the CDK1 promoter and that overexpression of c-MYC leads to increased expression of CDK1 [29–31]. Therefore, it is probable that TPA interferes with the PR transcriptional activation of c-MYC resulting in a plethora of downstream effects one of which is decreased CDK1 expression and no transit through G2/M. This is an hypothesis to be tested.

Progesterone binding to membrane-proximal PR activates cytoplasmic kinases that participate in signaling pathways that result in a number of post-translational modifications of PR [5]. These receptor modifications determine PR function by altering PR intracellular localization and turnover, and the specific promoters to which PR binds. PR-initiated rapid c-Src → MAPK signaling stimulates feed-forward phosphorylation of PR-B at serine 345, which tethers to Sp1 and increases the expression of both *EGFR* and *p21* [32]. Our data reveal that expression of both of these genes is significantly downregulated by TPA, which suggests that the antiprogestin binding may interfere, perhaps by altering PR conformation, with the phosphorylation of serine 345 or with the binding to Sp1.

Beato and colleagues have studied how nucleosomal organization and higher order chromatin structure influences the access of PR to its DNA binding sites [33]. Another of the consequences of activation of the c-SRC tyrosine kinase pathway is the phosphorylation of PR and Mitogen- and stress-activated protein kinase-1 (MSK1) by Extracellular signal-regulated protein kinases 1 and 2 (ERK1/2). The resulting ternary complex, pPR/pERK/pMSK1 is the active form of the receptor, which interacts with chromatin targets in a subset of target genes [34]. We observe significant downregulation of MSK1 gene expression by TPA, which may limit the formation of the ternary complex and therefore progesterone-mediated gene expression.

The protein encoded by *SMARCE1* (*BAF57*), a component of the SWI/SNF chromatin remodeling complex, plays an important role in ERα-mediated gene transcription and estrogen-stimulated proliferation [35] as well as being required for the response to androgen receptor (AR) agonists [36, 37]. In contract to the critical role BAF57 plays in regulating ER and AR function, transcription is significantly decreased by the progesterone agonist R5020 and increased by the addition of TPA to R5020. In addition, IPA upstream analysis indicted SMARCE1 activation in the TPA treated T47D cells (Additional file 4: Table S5). Transfection of the breast cancer cell line BT549 with *BAF57* results in cell cycle arrest and apoptosis [38]. This is another avenue for further investigation to determine if the increase in *SMARCE1 (BAF57*) following TPA treatment is responsible for the apoptosis observed at 24 h.

The cell cycle genes homology region CHR is a motif found in the promotors of many late cell cycle genes that display maximal expression in G2 and M [22]. This region is bound by the DREAM complex in G0 and early G1, which represses expression of the late cell cycle genes. In early S phase MMB binds to the CHR and later in the cell cycle MMB recruits FOXM1, which results in initiation of transcription of the late cell cycle genes. Proteosomal degradation of B-MYB in G2 and M leads to maximal expression of these genes through activation by FOXM1-MuvB (LIN9, LIN37, LIN52, LIN54 and RBBP4) [22]. Of these genes, only the expression of FOXM1 is affected by TPA and it is likely that the decreased expression of many of the 16 genes in the presence of TPA is due to the decreased binding of FOXM1 to CHR. CHR sites are usually found close to two to three CCAAT-box elements that bind the NFY transcription factor to activate transcription [39]. 14 of the 16 genes display the consensus biding sequence, however there was no statistically different expression of the NFY genes in the presence of TPA. Nonetheless, IPA upstream analysis suggests inhibition NFY mediated expression. Other factors, such as E2Fs, cooperate with NFY proteins to active transcription [39, 40]. The expression of E2F1 has been shown to be regulated by progestins [41] and, therefore, E2Fs are candidates for further study of the regulation of the 16 genes.

Our translational application of these observations is the development of predictive biomarkers, which is particularly challenging in the prevention setting. Preventive interventions must be based on an understanding of breast cancer risk and of how risk is transduced at the molecular level. Clinical and mouse data, reviewed by Ober and Edwards [42], indicate that the cell proliferative signaling pathways regulated by progesterone/PR contribute to the initiation and development of breast tumors. Based on epidemiologic data, Pike and colleagues proposed over three decades ago that an agent, which increases mitotic activity, such as progestogens, increases the probability of converting DNA damage (exogenous and endogenous) into mutations [9]. They estimated that the combined effect of a 2-year delay in menarche and a zero postmenopausal mitotic rate would reduce breast cancer incidence in the US by 50 %. Our data, which point to a significant effect of antiprogestins, such as TPA, on mitosis (Fig. 5c,d) and transit through the cell cycle, align nicely with this elegant preclinical and epidemiologic work, supporting the potential efficacy of these drugs in the prevention of breast cancer. The development of antiprogestins as breast cancer prevention agents requires the identification of biomarkers that reflect effective abrogation of progesterone signaling, particularly in pathways that are known to be involved in the evolution of malignancy. A strength of our approach is the comparison of genes with increased expression in T47D cells following R5020 administration with those genes with increased expression in the normal breast during the luteal phase, thereby identifying a gene set that predicts functional endogenous progesterone signaling in human breast tissue in the presence of endogenous estrogen. A subset of the initially identified 16 overlapping genes, AURKB, BUB1, CDC20, CDC25C, CDCA3, CDK1, CCNA2, CCNB2 and TPX2, were validated in a separate, independent experiment in which they had significantly increased expression in the presence of P4 and R5020 and significantly decreased expression when TPA was administered. These data enable us to define a potential biomarker set for trials where TPA is being tested for the prevention, and possibly the therapy, of human breast cancer. We envision that the expression of these genes could be assayed in high-risk premalignant lesions thereby identifying both the lesions whose growth is driven by progesterone signaling and the patients who would potentially benefit from TPA prevention. With regard to therapy, the ability of the genes we have identified, individually or in combination, to select sensitive tumors is presently being tested in a preclinical model; and will subsequently be tested in our ongoing clinical trial. Recent data of Mohammad and colleagues comparing the growth of MCF-7 xenograft tumors in the presence of estrogen alone or estrogen plus progesterone demonstrates decreased growth with the addition of progesterone [43]. This is similar to our data in Fig. 1g. Whether the addition of TPA will result in additional reduced proliferation that also mirrors our in vitro data is the purpose of our trial.

## Conclusions

TPA administration to T47D cells results in a decrease in cell proliferation at 24 h, which is maintained over time only in the presence of estradiol. One possible mechanism for this observed decrease is that TPA, by blocking progesterone signaling, decreases the expression of genes that facilitate the G2/M transition resulting in fewer cells in this phase of the cell cycle. Comparing genes induced by the progestin R5020 in T47D cells with those increased in the luteal-phase normal breast, we have identified a set of genes that predict functional progesterone signaling in tissue. This gene set may enable the identification of progesterone-responsive lesions and, thereby the selection of patients who will benefit from TPA utilized for the prevention, and possibly the therapy, of human breast cancer.

## Additional files

Additional file 1:
**Table S2.** Slope of lines in Fig. 1 d-f; **Table S3.** Genes involved in cell cycle progression; **Table S4.** Primers utilized for RT-qPCR validation of microarray results. (XLSX 16 kb) Additional file 2: Table S1. 2 way ANOVA, multiple comparisons with Bonferroni correction of proliferation data presented in Fig. 1. (XLS 308 kb) Additional file 3: Figure S1. RT-qPCR data from the technical validation of the array data. (PDF 133 kb) Additional file 4: Table S5. IPA Upstream Analysis, except where noted, data was chosen to be included if the gene was differentially expressed and the z-score significant; **Table S6.** Subset of the 16 genes with E2F1 and Myc binding in the MCF-7 Encode track; **Table S7.** Motif enrichment results; **Table S8.** Subset of 16 genes with CHR motif in regulatory region; **Table 9.** Subset of 16 genes with NFY motif in regulatory region; **Table 10.** Subset of 16 genes with evolutionary conserved CHR elements and bound by MMB complex and FOXM1 as determined by Müller et al. [22]. (XLS 105 KB) Additional file 5: Figure S2 MetaCore (Thompson Reuters) analysis of genes significantly differentially expressed in T47D cells in response to exposure to R5020 for A. 6 h, B. 24 h. (PPTX 1560 kb) Additional file 6: Figure S3 Ingenuity Pathway Analysis Upstream Analysis of differentially expressed genes: Effect of c-Myc activation/deactivation on gene expression. (PPTX 1503 kb)

## Abbreviations

AI: Aromatase Inhibitor
ANOVA: analysis of variance
BAF57: SWI/SNF related, matrix associated, actin dependent regulator of chromatin, subfamily e, member 1
CCND1: cyclin D1
CDK1: cyclin-dependent kinase 1
cDNA: complementary DNA
CHS: charcoal stripped
DMSO: dimethyl sulfoxide
DNA: deoxyribonucleic acid
E2: estradiol
EDTA: ethylenediaminetetraacetic acid
EGFR: epidermal growth factor receptor
ER: estrogen receptor
ERK1/2: extracellular signal-regulated protein kinases 1 and 2
FBS: fetal bovine serum
FDR: false discovery rate
GAPDH: glyceraldehyde 3-phosphate dehydrogenase
GR: glucocorticoid receptor
H: hours
MAPK: mitogen-activated protein kinase
mL: milliliter
mM: millimole
MPA: 17α-hydroxy-6α-methylprogesterone acetate
MSK1: mitogen- and stress-activated protein kinase-1
Ng: nanogram
nM: nanomolar
p21: CDKN1A (cyclin-dependent kinase inhibitor 1A (p21, Cip1))
P4: progesterone
PAGE: polyacrylamide gel electrophoresis
PBS: phosphate buffered saline
PI: propidium iodide
PR: progesterone receptor
PRE: progesterone response element
R5020: promegestone
RLU: renilla luciferase unit
RNA: ribonucleic acid
RNA-Seq: RNA sequencing
RT-qPCR: reverse transcription-quantitative polymerase chain reaction
RU-486: mifepristone
SDS: sodium dodecyl sulfate
SERM: selective estrogen receptor modulator
SMARCA1: SWI/SNF related, matrix associated, actin dependent regulator of chromatin, subfamily a, member 1
TPA: 17α-acetoxy-21 methoxy-11β[4-N,N-dimethylaminophenyl]-19-norpregna-4,9-diene-3,20-dione; telapristone acetate
X: fold
Μg: microgram
μM: micromolar
