# Supplementary material for: Progesterone receptor blockade in human breast cancer cells decreases cell cycle progression through G2/M by repressing G2/M genes
Source: BMC Cancer. 2016 May 23;16:326. doi: 10.1186/s12885-016-2355-5 (PMC4878043; doi:10.1186/s12885-016-2355-5)
Supplement: Additional file 3: Figure S1. — RT-qPCR data from the technical validation of the array data. (PDF 133 kb) [file 12885_2016_2355_MOESM3_ESM.pdf]

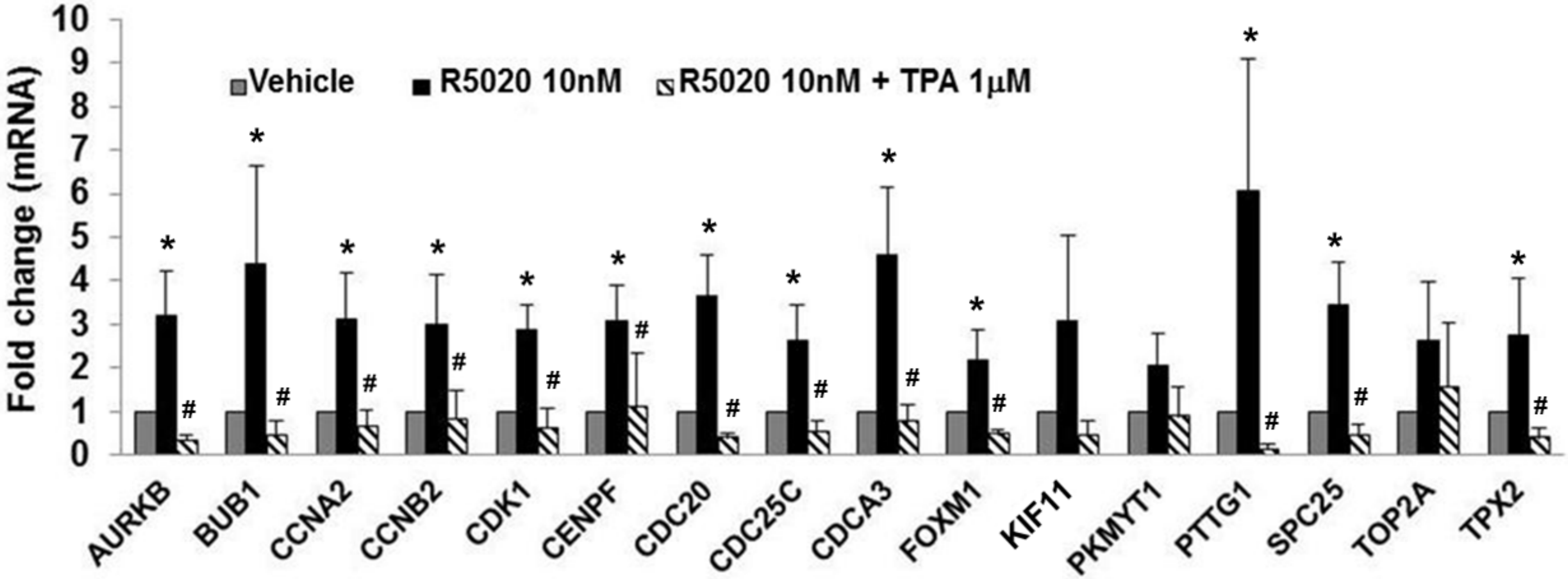

Supplemental Figure S1. Technical validation of array data. RT-qPCR data for the 16 genes using RNA of T47D cells that was utilized for the microarrays. 13 of the 16 genes are significantly upregulated in the Vehicle vs. R5020 cohort, *p< 0.05. KIF11, PKMYT1 and TOP2A were not able to be validated. The same 13 genes were downregulated # p<0.05 for R5020 vs. R5020 plus TPA.
