# Supplementary material for: Progesterone receptor blockade in human breast cancer cells decreases cell cycle progression through G2/M by repressing G2/M genes
Source: BMC Cancer. 2016 May 23;16:326. doi: 10.1186/s12885-016-2355-5 (PMC4878043; doi:10.1186/s12885-016-2355-5)
Supplement: Additional file 5: Figure S2 — MetaCore (Thompson Reuters) analysis of genes significantly differentially expressed in T47D cells in response to exposure to R5020 for A. 6 h, B. 24 h. (PPTX 1560 kb) [file 12885_2016_2355_MOESM5_ESM.pptx]

## Slide 1
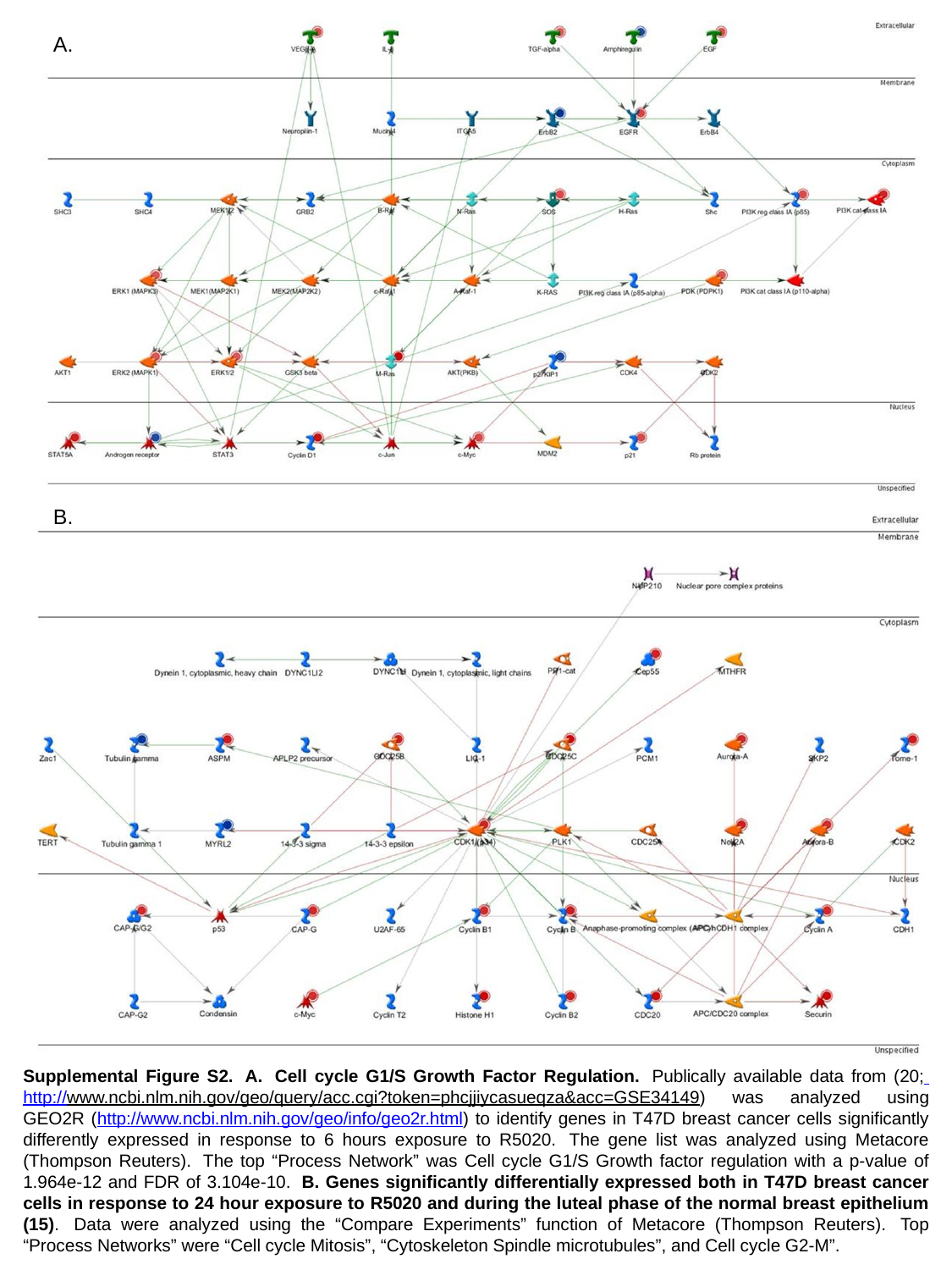

A.
B.
Supplemental Figure S2.  A.  Cell cycle G1/S Growth Factor Regulation.  Publically available data from (20; http://www.ncbi.nlm.nih.gov/geo/query/acc.cgi?token=phcjjiycasueqza&acc=GSE34149) was analyzed using GEO2R (http://www.ncbi.nlm.nih.gov/geo/info/geo2r.html) to identify genes in T47D breast cancer cells significantly differently expressed in response to 6 hours exposure to R5020.  The gene list was analyzed using Metacore (Thompson Reuters).  The top “Process Network” was Cell cycle G1/S Growth factor regulation with a p-value of 1.964e-12 and FDR of 3.104e-10.  B. Genes significantly differentially expressed both in T47D breast cancer cells in response to 24 hour exposure to R5020 and during the luteal phase of the normal breast epithelium (15).  Data were analyzed using the “Compare Experiments” function of Metacore (Thompson Reuters).  Top “Process Networks” were “Cell cycle Mitosis”, “Cytoskeleton Spindle microtubules”, and Cell cycle G2-M”.
