# Supplementary material for: Progesterone receptor blockade in human breast cancer cells decreases cell cycle progression through G2/M by repressing G2/M genes
Source: BMC Cancer. 2016 May 23;16:326. doi: 10.1186/s12885-016-2355-5 (PMC4878043; doi:10.1186/s12885-016-2355-5)
Supplement: Additional file 6: Figure S3 — Ingenuity Pathway Analysis Upstream Analysis of differentially expressed genes: Effect of c-Myc activation/deactivation on gene expression. (PPTX 1503 kb) [file 12885_2016_2355_MOESM6_ESM.pptx]

## Slide 1
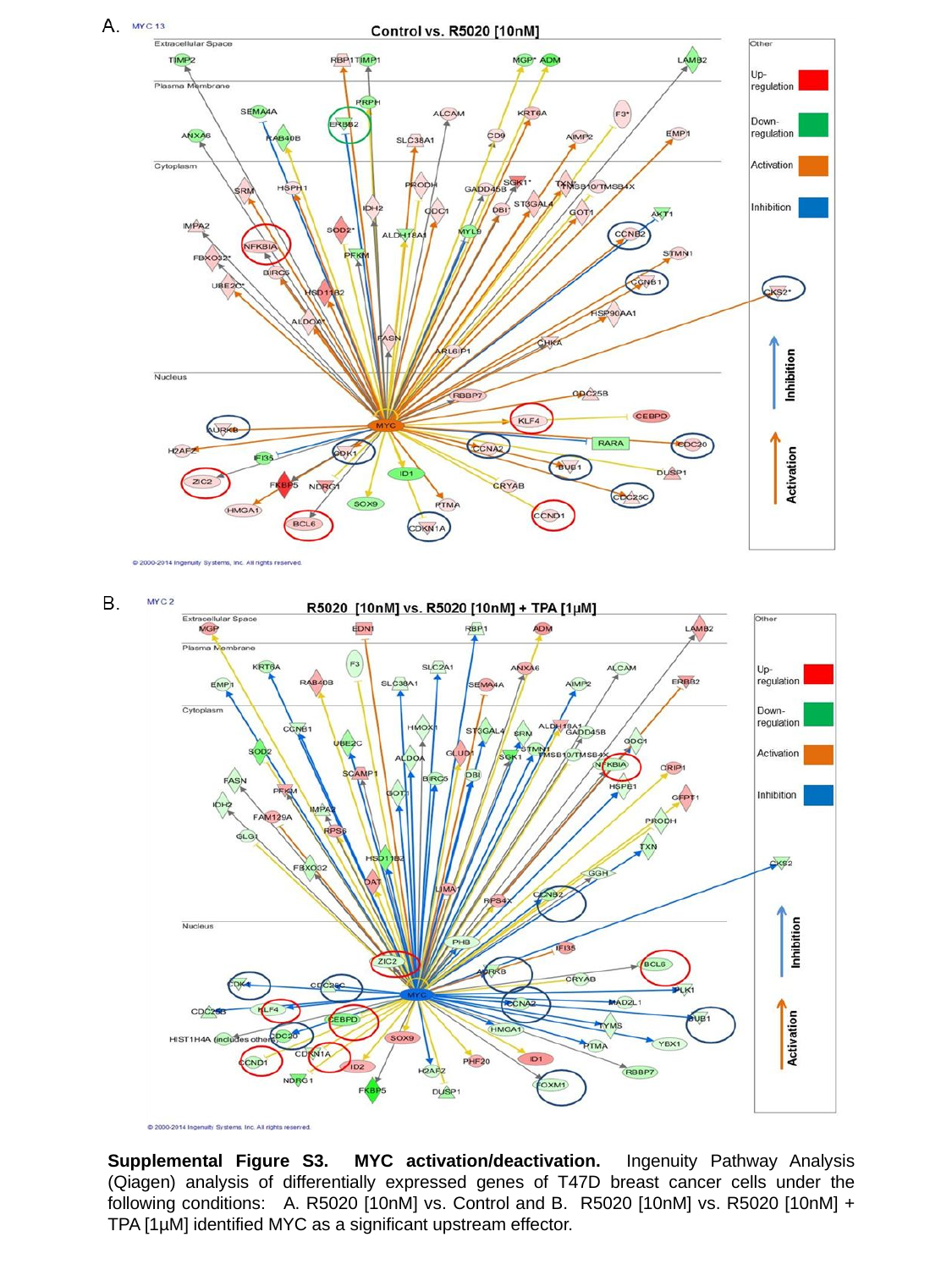

Supplemental Figure S3. MYC activation/deactivation. Ingenuity Pathway Analysis (Qiagen) analysis of differentially expressed genes of T47D breast cancer cells under the following conditions: A. R5020 [10nM] vs. Control and B. R5020 [10nM] vs. R5020 [10nM] + TPA [1µM] identified MYC as a significant upstream effector.
